# A new buffering theory of social support and psychological stress

**DOI:** 10.1371/journal.pone.0275364

**Published:** 2022-10-12

**Authors:** Stelios Bekiros, Hadi Jahanshahi, Jesus M. Munoz-Pacheco

**Affiliations:** 1 LSE Health Centre & Department of Health Policy, London School of Economics and Political Science (LSE), London, United Kingdom; 2 Faculty of Economics &Management (FEMA), University of Malta, Msida, Malta; 3 Department of Mechanical Engineering, University of Manitoba, Winnipeg, Canada; 4 Faculty of Electronics Sciences, Benemerita Universidad Autonoma de Puebla, Puebla, Mexico; Hodeidah University, YEMEN

## Abstract

A dynamical model linking stress, social support, and health has been recently proposed and numerically analyzed from a classical point of view of integer-order calculus. Although interesting observations have been obtained in this way, the present work conducts a fractional-order analysis of that model. Under a periodic forcing of an environmental stress variable, the perceived stress has been analyzed through bifurcation diagrams and two well-known metrics of entropy and complexity, such as spectral entropy and C0 complexity. The results obtained by numerical simulations have shown novel insights into how stress evolves with frequency and amplitude of the perturbation, as well as with initial conditions for the system variables. More precisely, it has been observed that stress can alternate between chaos, periodic oscillations, and stable behaviors as the fractional order varies. Moreover, the perturbation frequency has revealed a narrow interval for the chaotic oscillations, while its amplitude may present different values indicating a low sensitivity regarding chaos generation. Also, the perceived stress has been noted to be highly sensitive to initial conditions for the symptoms of stress-related ill-health and for the social support received from family and friends. This work opens new directions of research whereby fractional calculus might offer more insight into psychology, life sciences, mental disorders, and stress-free well-being.

## 1. Introduction

Stressful events are strongly connected to social factors [1]. Moreover, since the highly influential Cohen’s study [2], social support and its interaction with stress have been tightly tied to factors affecting health and well-being. More precisely, social support received from family and friends has proven to positively impact health by moderating the adverse effects of stress. In fact, the more social support an individual receives, the better overall mental and physical health he or she will have [3, 4]. In this respect, some studies also suggest that social support affects mental health rather than physical health, and those diverse kinds of support can have different effects on perceived stress [5, 6].

So far, a variety of mathematical models have been used to understand and predict real-world phenomena [7–14]. Thus, finding a model accurately describing psychological dynamics could pave the way for long and short-term predictions of mental diseases, as well as for designing appropriate therapies [15]. In other words, mathematical modeling aims to anticipate the dynamics of psychological systems and control them as effectively as possible concomitant diseases. Some seminar studies dealing with the modelling of psychological phenomena can be found in [16, 17].

So far, a lot of research has been done on psychological stress [18–20]. In [21], the effects of long-lasting psychological stress on social behaviours is investigated using a predator stress model. In [22], Kapasia et al. attempt to examine factors associated with psychological stress as well as academic satisfaction and future academic risk during the COVID-19 epidemic. In another study, various stressors related to covid-19, including risk exposure, limited medical treatment access, reduced income, and perceived discrimination, and their association with psychological distress were investigated [23]. They found that social support in the neighborhood can reduce psychological distress and buffer the effects of stressors. This is while the support of family and friends has a limited effect on coping with stress. Stress-buffering hypothesis has been introduced to interpret the effect of stress moderation [24]. This hypothesis states that stress moderation may happen by processes associated with the value of social support (main effects), as well as by processes associated with the value of social support and stress (which is called buffering effect). In other words, buffering represents the interaction of the stressor levels with the social support received from friends and family. Thus, when the level of stress increases, the buffering effect becomes more critical. This stress moderation hypothesis has provided a fruitful situation for the advent of more complex models that investigate the relationships between stress, support, and illness. Hence, psychological models considering factors that are related to the buffering effect have attracted considerable attention in the last years [25, 26].

As a matter of fact, the development of trustable tools is the most critical challenge in the modeling of real-world, practical systems [27–31]. To reach this goal, fractional calculus has been recently proposed as a useful alternative [32, 33]. Indeed, fractional calculus is an excellent tool for the description of hereditary properties and memory of systems, which has been applied in a wide variety of scenarios in the last years [34–38]. Also, recently, the application of fractional calculus in social events and even disease dynamics have attracted a lot of attention. In [39–41] various fractional-order models of the transmission dynamics of COVID-19 have been proposed and studied.

In [42], a fractional-order dynamic model of love has been examined and its chaotic behaviour has been studied by investigating different orders. Additionally, in [43], the rich dynamics of a fractional-order love system with the fractional order derivative and model parameters have been studied. Furthermore, the control problem has been theoretically investigated. In [44], through fractional-order differential equations, dynamical model of happiness has been studied. By classifying persons of different personalities and impact factors of memory (IFM) with a distinct set of model parameters, it has been illustrated that such fractional-order models might display multiple behaviors with and without external situations. In [45], chaos control and dynamical synchronization model of happiness with fractional order have been studied.

Nonetheless, no study has used these fractional techniques to investigate the influence of social support buffering on stress. Despite the fact that fractional calculus gives a helpful and practical viewpoint in the modeling of real-world systems, publications that employ fractional calculus to describe psychological dynamics are rare. As a result, there is still an opportunity for improvement in nonlinear models that take into account the impacts of social support buffering on physiological stress. Motivated by this, the current study introduces for the first time a fractional-order analysis of a previously published dynamic model of stress-related processes.

The suggested modelling, which provides a generalization dynamic of social support and psychological stress, is justified by the fact that the time evolution of psychological interactions, like other fractional systems, is inherently affected by memory. The fractional-order model is introduced, and its parameters are delineated. Then, in addition to bifurcation diagrams, analyses of entropy and complexity are also conducted in terms of spectral entropy and C_0_ complexity. Considering a periodic variation in environmental stress, as well as a self-kindling in the subject’s stress response, new and interesting insights into the relationships between stress, social support, and health are obtained. For instance, it is shown that stress experienced by an individual is strongly affected by initial conditions for physical or mental symptoms, as well as for the received social support.

The rest of the study is planned as follows. Section 2 describes the mathematical model of socially buffered stress processes and some basic concepts about fractional calculus. Afterward, the fractional-order model of the socially buffered stress processes is proposed as a new approach, and some of its properties are delineated. Section 3 outlines entropy and complexity analyses conducted on the resulting time series from the proposed approach. Finally, the main conclusions of the present study are summarized in Section 4.

## 2. Stress-buffering hypothesis

The socially buffered response model can be expressed as follows [46, 47]:

$$\frac{dX(t)}{dt}=k_{1}A+(k_{2}B-k_{6})X(t)+k_{3}Y(t)-k_{4}X(t)Z(t)-k_{5}X^{2}(t),$$ (1)

$$\frac{dY(t)}{dt}=k_{6}X(t)-k_{7}Y(t),$$ (2)

$$\frac{dZ(t)}{dt}=k_{8}(S_{0}-Z(t))Y(t)-\beta k_{4}X(t)Z(t)-k_{9}Z(t),$$ (3)

where *X*(*t*) denotes the perceived psychological stress, including factors such as the person’s cognitive appraisal [48], and the perceived psychological stress measured by observed stressors, self-reported, taxing life events, or hassles regarding an event [49]. The variable *Y*(*t*) indicates the symptoms of stress-related ill-health, which can obtain through diverse clinical instruments. Moreover, *Z*(*t*) stands for the received social support. This variable can be estimated via the received emotional and tangible assistance, the number of involved family members and friends, and so forth. In this model, it is also supposed that there exists bounded social support. Therefore, *S*_0_ = *Z*+*U*, where *Z* and *U* respectively indicate the social support currently in use and the social support existing at the present time but not involved in buffering stress. Consequently, *S*_0_ indicates the total available social support. The rate parameters *k*_*i*_ (*for i* = 1,…,9) depend on the personality and features of the individual under stress. A and B respectively refer to the theoretical and experimental measures of environmental stress. More precisely, A indicates the environmental circumstances that directly cause a raising of the stress response, and B refers to the events and circumstances where the growth of stress happens due to the existence of stress itself. By fitting the model, all weights can be estimated for every particular individual and environment [46].

The model is quite similar to the one presented by Oregonator [50], which is a successful model of the oscillatory Belousov–Zhabotinsky chemical reaction. The matching of the model with real-world results has been clearly demonstrated by Epstein and Pojman [51]. Also, it has been shown that selecting suitable values for the described parameters results in psychologically reasonable outcomes, thus exhibiting oscillation, multiple stationary states, spatial pattern formation, bursting, and chaos [46]. Moreover, for most parameters, states of the system exhibit unchanging stationary values, suggesting that the model converges to “normal” dynamics of stress and symptoms of illness, and moreover, demonstrating recent interpretations of homeostasis [47].

Field and Schuldberg [47] have conducted a search of parameters on the presented model, which demonstrated that the model (Eqs (1)–(3)) does not show chaotic behavior in support seeking and stress levels. However, when the environmental stress value *A* changes periodically, chaos was detected for some conditions [46]. To this end, the following pattern for *A* was considered:

$$A=A_{0}+\rho sin\;(\omega t),$$ (4)

where *ω* and *ρ* denote the angular frequency and amplitude of the modulation, respectively. The magnitude and frequency of stress are two key factors that affect the system stability. Thus, when these parameters increase, the person experiences a more extensive and more rapid incidence of stressors. Also, high levels of stress over an extended period of time result in “complex trauma” [52]. In general terms, such conditions can bring new diagnostic categories and trauma disorders [53]. In what follows, we propose and investigate a pioneering fractional-order model of the socially buffered stress processes.

### 2.1. Fractional-order modeling

Fractional-order derivatives have been used widely, not only in an artificial way, but also with real-world physical phenomena [54]. They have been pointed out as valuable tools for understanding biological phenomena. In this way, a fractional form of the system described in Eqs (1)–(3) can be expressed as follows:

$${{}_{t}}_{0}^{C}D_{t}^{q_{1}}X(t)=k_{1}A+(k_{2}B-k_{6})X(t)+k_{3}Y(t)-k_{4}X(t)Z(t)-k_{5}X^{2}(t),$$ (5)

$${{}_{t}}_{0}^{C}D_{t}^{q_{1}}Y(t)=k_{6}X(t)-k_{7}Y(t),$$ (6)

$${{}_{t}}_{0}^{C}D_{t}^{q_{1}}Z(t)=k_{8}(S_{0}-Z(t))Y(t)-\beta k_{4}X(t)Z(t)-k_{9}Z(t),$$ (7)

where *A* = *A*_0_+*ρ* sin(*ωt*), and the values selected for the system parameters are listed in Table 1. These values were taken from [2].

**Table 1:** Values selected for the system parameters.

Parameter	*A*_0_	*S*_0_	*β*	*k*_1_	*k*_2_	*k*_3_	*k*_4_	*k*_5_	*k*_6_	*k*_7_	*k*_8_	*k*_9_
**Value**	1	10	0.5	1	1	0.01	2	0.3	0.01	0.01	0.1	0.01

It is well-known that fractional calculus is a generalization of differentiation and integration of non-integer order, defined by a continuous integro-differential operator ${{}_{t}}_{0}^{C}D_{t}^{q_{1}}$, where α and t are the limits of the operation and *q*∈ℝ. In the literature, there are many definitions for the integro-differential operator. However, all are not adequate to define whether an operator can be considered as fractional. An excellent work published by Ortigueria et al. addresses this challenge by analyzing several definitions of fractional derivatives [55]. An operator is considered as a fractional derivative when it mandatorily satisfies the criteria formulated by Ortigueira and Machado [56, 57].

#### 2.1.1. Introducing the Caputo fractional operator

The Caputo fractional derivative was selected in the present study because it has been revisited and analyzed under wide sense criterion and strict sense criterion, then fulfilling properties such as linearity, identity, backward compatibility, index law, and generalized Leibniz rule [56, 57]. Additionally, another feature motivating the use of the Caputo operator is related to the differentiation of a constant function, because ${{}_{t}}_{0}^{C}D_{t}^{q}k=0$ where *k*∈ℝ. Moreover, unlike fractional differential equations with Riemann-Liouville derivative, which are initialized by derivatives of fractional-order, an initial value problem for fractional differential equations with Caputo’s derivatives can be formulated as in ordinary differential equations.

**Definition 1 [58]:** The Caputo fractional derivative with order *q*>1 for a function *f*(*t*)∈ℂ^*n*^([*t*_0_,∞), ℝ) is defined

$${{}_{t}}_{0}^{C}D_{t}^{q}f(t)=\frac{1}{\Gamma (n-q)}\int_{t_{0}}^{t}\frac{f^{\left(n\right)}(s)}{{\left(t-s\right)}^{q-n+1}}ds$$ (8A)

on the interval [*t*_0_, *t*]. When 0<*q*<1 is given as follows

$${{}_{t}}_{0}^{C}D_{t}^{q}f(t)=\frac{1}{\Gamma (1-q)\int_{t_{0}}^{t}\frac{f^{\prime }(s)}{{\left(t-s\right)}^{q}}ds}.$$ (8B)

When ($t_{0}^{c}D_{t}^{1}=D$, we recover the standard integer-order derivative whereas ${{}_{t}}_{0}^{C}D_{t}^{0}=I$ is the identity operator. Using the fractional-order operator of the Caputo derivative in Eq (8), we get the proposed fractional-order socially buffered response model as follows

$${{}_{t}}_{0}^{C}D_{t}^{q_{1}}X=k_{1}A+(k_{2}B-k_{6})X+k_{3}Y-k_{4}XZ-k_{5}X^{2},$$ (9)

$${{}_{t}}_{0}^{C}D_{t}^{q_{2}}Y=k_{6}X-k_{7}Y,$$ (10)

$${{}_{t}}_{0}^{C}D_{t}^{q_{3}}Z=k_{8}(S_{0}-Z)Y-\beta k_{4}XZ-k_{9}Z.$$ (11)

Based on the predictor-corrector Adams-Bashforth-Moulton (ABM) integrator, which relies on the analytical property that the initial value problem ${}_{a}D_{t}^{q}y(x)=f(x,y(x))$ is equivalent to the Volterra integral [59], we can obtain the discretized form of the system as follows:

$$X_{n+1}(t)=X_{0}+\frac{h^{q_{1}}}{\Gamma (q_{1}+2)}(f_{1}(X_{n+1}^{p},Y_{n+1}^{p},Z_{n+1}^{p})+\sum_{j=0}^{n}\alpha_{1,j,n+1}f_{1}(X_{j},Y_{j},Z_{j})),$$ (12)

$$Y_{n+1}(t)=Y_{0}+\frac{h^{q_{2}}}{\Gamma (q_{2}+2)}(f_{2}(X_{n+1}^{p},Y_{n+1}^{p},Z_{n+1}^{p})+\sum_{j=0}^{n}\alpha_{2,j,n+1}f_{2}(X_{j},Y_{j},Z_{j})),$$ (13)

$$Z_{n+1}(t)=Z_{0}+\frac{h^{q_{3}}}{\Gamma (q_{3}+2)}(f_{3}(X_{n+1}^{p},Y_{n+1}^{p},Z_{n+1}^{p})+\sum_{j=0}^{n}\alpha_{3,j,n+1}f_{3}(X_{j},Y_{j},Z_{j})),$$ (14)

where

$$X_{n+1}^{p}(t)=X_{0}+\frac{1}{\Gamma (q_{1})}\sum_{j=0}^{n}\beta_{1,j,n+1}f_{1}(X_{j},Y_{j},Z_{j}),$$ (15)

$$Y_{n+1}^{p}(t)=Y_{0}+\frac{1}{\Gamma (q_{2})}\sum_{j=0}^{n}\beta_{2,j,n+1}f_{2}(X_{j},Y_{j},Z_{j}),$$ (16)

$$Z_{n+1}^{p}(t)=Z_{0}+\frac{1}{\Gamma (q_{3})}\sum_{j=0}^{n}\beta_{3,j,n+1}f_{3}(X_{j},Y_{j},Z_{j}),$$ (17)

with

$$\alpha_{i,j,n+1}=\left\{ \begin{array}{c} n^{q_{i}+1}-(n-q_{i}){\left(n+1\right)}^{q_{i}},j=0,\\ {\left(n-j+2\right)}^{q_{i}+1}+{\left(n-j\right)}^{q_{i}+1}-2{\left(n-j+1\right)}^{q+1},\\ 1, \end{array}\right.\begin{array}{c} j=0\\ 1\leq j\leq n\\ j=n+1 \end{array}$$

and

$$\beta_{i,j,n+1}=\frac{h^{q_{i}}}{q_{i}}({\left(n+1-j\right)}_{i}^{q}-{\left(n-j\right)}_{i}^{q}).$$

Here, *X*_0_, *Y*_0_, *Z*_0_, are the initial conditions, and *f*_1_, *f*_2_, and *f*_3_ are the right-hand sides of Eqs (5)–(7) as $f_{1}(X,Y,Z)=k_{1}A+(k_{2}B-k_{6})X+k_{3}Y-k_{4}XZ-k_{5}X^{2},\;f_{2}(X,Y,Z)=k_{6}X-k_{7}Y$, and $f_{3}(X,Y,Z)=k_{8}(S_{0}-Z)Y-\beta k_{4}XZ-k_{9}Z$. If *q*_*i*_ = *q*, for *i* = 1,2,3, the system is said to be commensurate and the convergence order is described as $|Y(t_{n})-Y_{n}|=O(h^{min(2,1+q)}),h\to 0$.

### 2.2. Equilibrium points of the stationary states

Setting ${{}_{t}}_{0}^{C}D_{t}^{q}=f(X,Y,Z;t)=0$ in Eqs (9–11) yields the equilibrium points *E*_*i*_ = (X*, Y*, Z*), which are *E*_1_ = (2.58, 2.58,0.90), *E*_2_ = (−0.009, −0.009, −54.8), and *E*_3_ = (−1.31, −1.31,0.91). It can be observed that only the stationary state *E*_1_ has positive values for *X*, *Y*, *Z*. Therefore, it represents an individual’s unchanging but dynamic state, i.e., a “dynamic equilibrium”. In this case, the stable stationary states can represent regions of generalized homeostasis, where some degree of dynamic constancy exists and can be sustained even under perturbation. By linearizing the system described in Eqs (9–11), we can study the stability of the stationary state *E*_1_ when the parameter *B* is varied. This parameter is important because it is considered as a psychological variable representing how much stress tends to build more stress. This phenomenon is sometimes referred to as “kindling.”

**Lemma 1:** *The equilibrium point E*_1_ *of the fractional-order socially buffered response model described in Eqs (*9–11) with *B*∈[1,2.2] and the parameters included in Table 1 is unstable for all *q*∈[0, 1) [60].

*Proof*. The characteristic equation of the fractional-order socially buffered response model is described in Eqs (9–11) at equilibrium point (2.58, 2.58,0.90) is given as λ^3^+4.016λ^2^−1.306λ+0.033 = 0. Here, λ_2,3_ are positive, real numbers. Therefore, the equilibrium point *E*_1_, considering *B*∈[1,2.2], is unstable for all *q* ∈ [0, 1].

### 2.3. The chaotic effect of memory in stress-related fractional-order social buffering

Regarding the previously presented integer-order version of the socially buffered response model, we have added the effect of memory in the mathematical model to analyze people’s stress-related processes. As well-known, if two Markovian processes (integer-order) start at two different times, the evolution of both processes is identical. However, the scenario is completely different for a non-Markovian approach (fractional-order), in which the memory plays the main role [61, 62]. In the proposed model, the strength (through the “length”) of the memory is controlled by the fractional-order. As *q* tends to 0, the influence of memory increases and vice-versa. It is important to point out that we only analyze the commensurate case, i.e., all the fractional orders are identical. For the scenario whereby incommensurate orders are needed, the stability analysis in section 2.2 should be derived using Theorem 4.6, page 79, given in [60]. In the commensurate framework, we hypothesize that the perceived psychological stress, *X*(*t*), should change as time evolves. For instance, the perceived stress could be lower when a person faces taxing life events at early stages than when he or she is initially stressed. Indeed, the symptoms of stress-related ill-health, *Y*(*t*), may get worse due to the accumulative stress effects. Finally, the received social support, *Z*(*t*), could reduce the stress impact as time evolves. It means the support has no immediate results, but as the person continues receiving such support (memory), it could be more motivated, and then the stress effects could be reduced.

**Fig 1:**
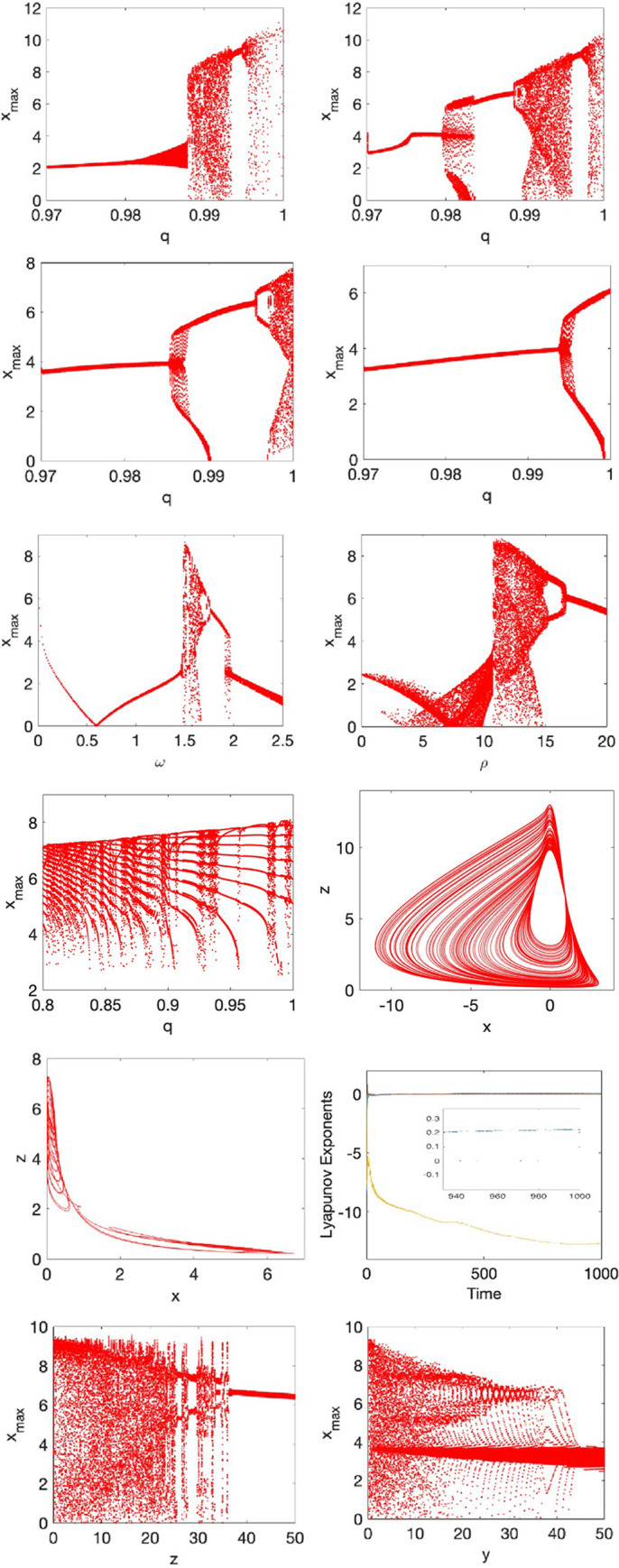
Nonlinear behaviors of the fractional-order stress system. Bifurcation diagrams as a function of the fractional-order q with *ρ* = 11, *ω* = 1.5, for a) B = 2.2, b) B = 2.0, c) B = 1.5, and d) B = 1.0. e) Bifurcation diagram for *ω* with q = 0.99, *ρ* = 11, and B = 2.2. f) Bifurcation diagram for *ρ* with q = 0.99, *ω* = 1.5, and B = 2.2. g) Bifurcation diagram varying q with *ρ* = 1, B = 2.2 and *ω* = 0.45. h) Chaotic attractor obtained from parameters in the subfigure a) when q = 0.99. i) Chaotic attractor obtained from parameters in the subfigure a) when q = 0. 98. j) Lyapunov exponents for the chaotic attractor in subfigure h) resulting in LE1 = 0.203, LE2 = 0, LE3 = -12.707 (magnified area from 940s to 1000s). k) and l) k) and l) Bifurcation diagrams showing the switching between chaos and stable dynamics.

Within the described context, the parameter of the environmental stress *A* is represented as a periodic forcing, and chaos behavior can then be founded as a function of the fractional-order. In this case, we set *A* = *A*_0_+*ρ* sin *ω t*, where *A*_0_ = 1, and the parameters *ρ* and *ω* are the amplitude and angular frequency of the modulation, respectively. We have conducted several numerical simulations under the external perturbation using the algorithm described in Eqs (12–14). Fig 1A–1D show the bifurcation diagrams for *B* = 2.2, *B* = 2.0, *B* = 1.5, and *B* = 1.0, respectively, and for different values of the fractional-order q. As can be seen, the fractional-order q can be considered as a control parameter for the chaos behavior. Therefore, the stress model can alternate between chaos, periodic oscillations, and stable behaviors as the fractional-order q varies. Fig 1E and 1F present the behavior of the force of the periodic perturbation and its frequency when q = 0.99. In this case, the perturbation frequency *ω* has a narrow interval for the chaotic oscillations, while the force q may set with different values indicating a low sensitivity regarding chaos generation.

An interesting result was also found when the fractional-order q varies in the range (0.8,1), then originating complex stability regions characterized by multiple alternations between periodic and chaotic oscillations, such as Fig 1G displays. The chaotic and periodic attractors are illustrated in Fig 1H and 1I, when *q* = 0.99 and *q* = 0.98, respectively. The validation of the chaotic oscillations in the fractional-order stress model was performed by the Lyapunov exponent method. For the case of chaotic behavior, the Lyapunov spectrum was LE1 = 0.203, LE2 = 0, and LE3 = −12.707 by applying the Benettin–Wolf algorithm modeled by Caputo’s derivative [63]. The Dimension Kaplan-Yorke is DKY = 2.984, which is computed by considering the definition 1 presented in [64]. Finally, the multi-stability in the fractional-order system for different values of arbitrary initial conditions for Y and *Z*, respectively, was also discovered. This result suggests that stress analyzed with the proposed fractional-order social support buffered response model is affected not only by the system parameters, but also by the initial conditions. More specifically, Fig 1K and 1L show switching between chaos and stable dynamics, thus highlighting that the psychological stress perceived by an individual can be affected by the initial conditions for the symptoms of stress-related ill-health, as well as for the initial conditions of the received social support.

It is worth noting that bifurcation diagrams were also conducted for *q* in (0,0.8); however, the results are not shown since they tend to either periodic behaviors or unbounded solutions, respectively. In this manner, we focus on the interval *q*∈[0.8,1] as given in Fig 1(A)–1(D) and Fig 1(G). This finding agrees with the literature on fractional-order systems, where the fractional orders for detecting chaos phenomena are typically q≥ 0.8 [65].

## 3. Complex nonlinear dynamics

The study of complex systems is often addressed by characterizing their resulting empirical time series in search of patterns and laws that rule their main dynamics. A variety of measures of entropy, relative entropy, complexity, fractal dimensions, etc., have been used for that purpose [66, 67]. In general terms, these metrics can be divided into two groups, i.e., those estimating the global structure of a time series, and those quantifying its time behavior. Whereas the former measure entropy or complexity of a sequence through its spectral distribution, the latter estimate regularity or predictability of a time series by analyzing its time distribution of samples. Although both kinds of indices have reported interesting results in diverse scenarios [68, 69], those based on the spectral transformation of the data provide a global statistical significance and then an easier interpretation. Indeed, these metrics analyze global energy features of a time series without paying special attention to specific local sequences [70]. Two common indices within this group are spectral entropy (SE) and *C*_0_ complexity. Both metrics have been used here to characterize the system described in Eqs (9–11).

From a mathematical point of view, given a time series of *N* samples in length, i.e., x(n)={x(0),x(1),…,x(N−1)}, computation of both SE and *C*_0_ complexity starts by removing its current part, such that

$$x(n)=x(n)-\bar{x},$$ (18)

where $\bar{x}=\frac{1}{N}\sum_{n=0}^{N-1}x(n)$. Then, its Fourier transform is computed as

$$X(k)=\sum_{n=0}^{N-1}x(n)e^{-j\frac{2\pi nk}{N}},$$ (19)

where *k* = 0, 1,…,*N*−1, and $j=\sqrt{-1}$ is the unit imaginary. Next, for SE estimation, the relative power spectral density of *x*(*n*) is obtained as

$$P(k)=\frac{{\left|X(k)\right|}^{2}}{\sum_{k=0}^{\frac{N}{2}-1}{\left|X(k)\right|}^{2}},$$ (20)

Consequently, $\sum_{k=0}^{\frac{N}{2}-1}P(k)=1$. Then, SE is yielded by computing Shannon Entropy from the resulting spectral density [71], i.e.,

$$SE(x,N)=-\frac{1}{\ln\left(\frac{N}{2}\right)}∙\sum_{k=0}^{\frac{N}{2}-1}P(k)∙ln(P(k)).$$ (21)

It should be noted that SE is normalized by its highest value, i.e. $\ln\left(\frac{N}{2}\right)$, to range from 0 to 1. The metric quantifies the flatness of the frequency spectrum, such that a high value suggests a flat, uniform spectrum with a broad spectral content, and a low value involves a spectrum with all the power condensed into a single frequency bin, i.e., a less complex, more predictable signal [72].

On the other hand, to compute *C*_0_ complexity, *X*(*k*) is modified according to a threshold obtained from the mean spectral power of *x*(*n*) [73]. More precisely, that threshold is obtained as

$$G_{N}=\frac{2∙r}{N}\sum_{k=0}^{\frac{N}{2}-1}{\left|X(k)\right|}^{2},$$ (22)

where *r* is a control parameter. Then, the spectral distribution of *x*(*n*) is redefined as

$$\tilde{X}(k)=\left\{ \begin{array}{cc} X(k), & if\;{\left|X(k)\right|}^{2}>G_{N},\\ 0, & if\;{\left|X(k)\right|}^{2}\leq G_{N}, \end{array}\right.$$ (23)

and the inverse Fourier transform can be used to obtain $\tilde{x}(n)$, i.e.

$$\tilde{x}(n)=\frac{1}{N}\sum_{k=0}^{N-1}\tilde{X}(k)\;e^{j\frac{2\pi nk}{N}},$$ (24)

where *n* = 0, 1,…,*N*−1. This new time series keeps the regular part of *x*(*n*), and then *C*_0_ complexity can be estimated through the ratio between the irregular part of *x*(*n*) and the original signal [73], i.e.,

$$C_{0}(x,r,N)=\frac{\sum_{n=0}^{N-1}{\left|x(n)-\tilde{x}(n)\right|}^{2}}{\sum_{n=0}^{N-1}{\left|x(n)\right|}^{2}}.$$ (25)

Clearly, the larger the value of *C*_0_, the larger the complexity of *x*(*n*) [73].

**Fig 2:**
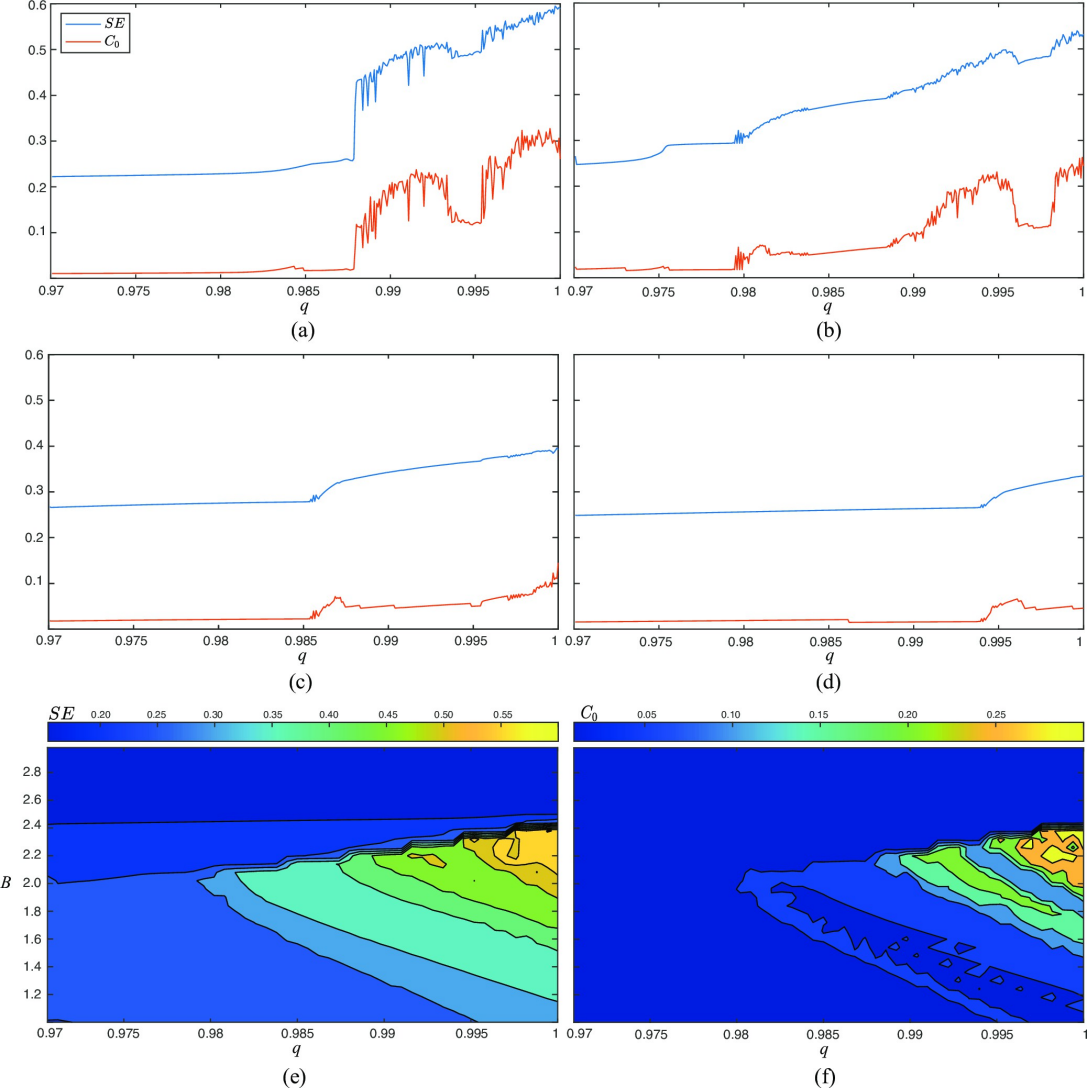
Variation of SE and *C*_0_ as a function of *B* and *q* for several scenarios. In all cases, SE and *C*_0_ were computed from the variable *x*(*n*) and the parameters *ρ* = 11 and *ω* = 1.5 were used. In the first four panels the parameter *B* took values of (a) 2.2, (b) 2.0, (c) 1.5, and (d) 1.0. The two last panels show contour plots for (e) SE, and (f) *C*_0_, when values of *B* and *q* range from 1 to 3, and from 0.97 to 1, respectively.

In the present work, both SE and *C*_0_ have been computed from the variable *x*(*n*) obtained for the system described in Eqs (9–11) by considering the conditions analyzed in the previous section. Thus, Fig 2 shows how dynamics change under the perturbation described in Eq (21) with *ω* = 1.5, *ρ* = 11, and different values of *B* and fractional orders q. As can be seen, the results presented by both metrics in Fig 2(A)–2(D) agree with those displayed by Fig 1(A)–1(D), thus clearly discerning between stable and chaotic behaviors. In fact, values of SE and *C*_0_ about 0.4 and 0.12 successfully discriminate between both states. Moreover, according to these thresholds, Fig 2(E) and 2(F) only exhibit chaos for a limited region, defined by values of B between 1.5 and 2.3 and fractional orders q between 0.9875 and 1. Nonetheless, it should be noted that the area showing chaos is wider when q increases.

**Fig 3:**
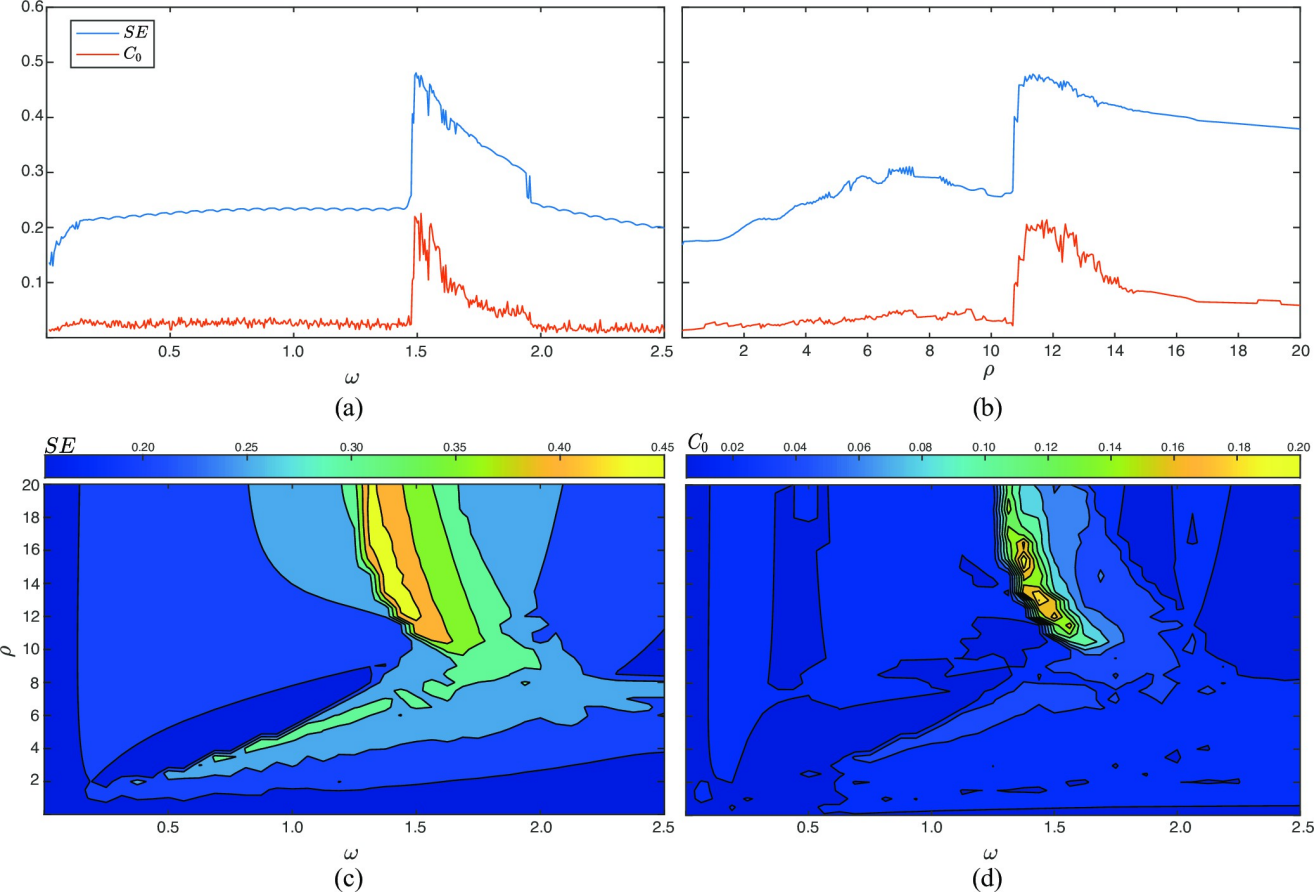
Variation of SE and *C*_0_ as a function of *ω* and *ρ* for several scenarios. In all cases, SE and *C*_0_ were computed from the variable *x*(*n*) and the parameters *B* = 2.2 and the fractional order *q* = 0.99 were used. In the first two panels the parameters (a) *ω* and (b) *ρ* were varied when *ρ* = 11 and *ω* = 1.5, respectively. The two last panels show contour plots for (c) SE, and (d) *C*_0_, when values of *ρ* and *ω* range from 0 to 20, and from 0 to 2.5, respectively.

On the other hand, Fig 3 shows the variation of SE and *C*_0_ as a function of both the force (ρ) and the frequency (*ω*) of the perturbation, when the fractional order q is set to 0.99. As can be seen in Fig 3(A) and 3(B), in both cases, the evolution is consistent with the bifurcation diagrams presented in Fig 1(E) and 1(F). Indeed, when ρ is fixed to 11, chaotic behavior is only noticed when *ω* ranges from 1.5 to 1.7 (see Fig 3(A)). Similarly, when *ω* is established to 1.5, stable behavior is observed for most values of ρ, apart from those between 11 and 14 (see Fig 3(B)). According to these findings, Fig 3(C) and 3(D) also display a narrow area where the system exhibits chaotic behavior. This region is roughly delimited by values of *ω* between 1.28 and 1.65, and values of ρ between 10.5 and 20.

**Fig 4:**
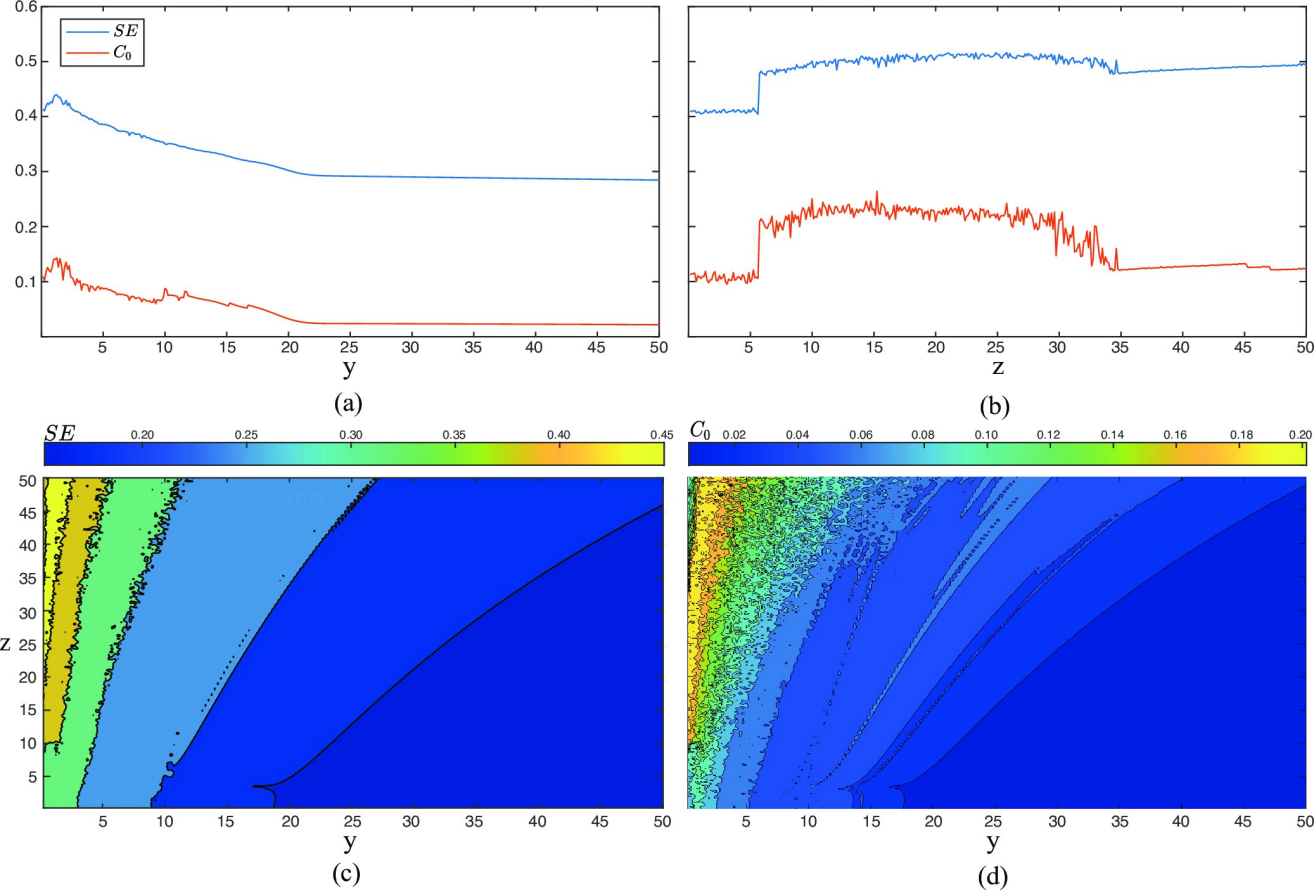
**Variation of SE and *C***_**0**_ **as a function of the initial conditions of *y* and *z*.** In all cases, SE and *C*_0_ were computed from the variable *x*(*n*) and the parameters *q* = 0.99, *ρ* = 11, and *ω* = 1.5 were used. In the first two panels the values of initial conditions of (a) *y* and (b) *z* were ranged from 0 to 50, whereas in the two last panels contour plots for (c) SE, and (d) *C*_0_ are shown.

Regarding the different initial conditions tested for Y and Z, values of SE and *C*_0_ displayed by Fig 4(A) and 4(B) were also in agreement with the bifurcation diagrams shown by Fig 1(k) and 1(l), thus presenting a multi-stable behavior of the system. Thus, chaotic behavior is only observed for a narrow range of initial values of Y from 0 to 2.5, approximately. Contrarily, a broader range of initial conditions of Z from 6 to 35 exhibit chaos. According to these results, contour plots of SE and *C*_0_ displayed in Fig 4(C) and 4(D) also present a chaotic region limited by a few initial values of Y (between 0 and 7) and most values of Z (between 0 and 50). Nonetheless, it should be noted that the larger the initial value of Z, the wider the region of initial values of Y exhibiting chaos.

Finally, it is worth noting that in all conducted analyses of SE and *C*_0_ have reported similar results. Nonetheless, *C*_0_ has proven to have a slightly higher sensitivity to small changes in the system dynamics, thus better discerning between chaotic and stable behaviors.

## 4. Conclusions

A fractional-order analysis of a dynamic system modelling people’s stress-related processes has been for the first time conducted in the present work. Thus, the stress perceived by an individual under a periodic environmental perturbation has been widely analyzed, and some novel insights have been obtained about how this emotional state relates to external stressors and social support. More precisely, the subject’s behavior has proven to be unstable and evolve from a stable stage to chaos for a narrow set of frequency and amplitude values of the external perturbation, when different fractional orders were analyzed. Moreover, our numerical simulations have also conspicuously confirmed that, in some cases, a small deviation in derivative order could result in a completely different dynamical behavior of the system. This finding has been previously unseen since assuming derivative orders to have only integer values restricts simulations and predictions to a limited manner. On the other hand, it has also been shown that not only the system parameters affect stress analyzed with the fractional-order social support buffered response model, but also the initial conditions could considerably affect it. These findings could be helpful in better understanding how an individual reacts to different levels of stressors and social-support recruitment and then taking preventive measures to avoid further health problems. Overall, this study is pioneering research in using fractional calculus for the analysis of stress, which has demonstrated the importance of considering a fractional framework for such kind of physiological model. As future work, the case where the memory contributions are distinct, i.e., incommensurate fractional orders, could be analyzed.
